# Autoregulation in Infants with Neonatal Encephalopathy

**DOI:** 10.15844/pedneurbriefs-29-10-2

**Published:** 2015-10

**Authors:** Andrea C. Pardo

**Affiliations:** 1 Division of Neurology, Ann & Robert H. Lurie Children’s Hospital of Chicago, Chicago, IL; 2 Departments of Pediatrics and Neurology, Northwestern University Feinberg School of Medicine, Chicago, IL

**Keywords:** Hypoxic Ischemic Encephalopathy, Therapeutic Hypothermia, Autoregulation

## Abstract

Researchers from Johns Hopkins School of Medicine report a pilot observational study investigating the influence of hemodynamic management in 28 neonates with hypoxic ischemic injury (HIE).

Researchers from Johns Hopkins School of Medicine report a pilot observational study investigating the influence of hemodynamic management in 28 neonates with hypoxic ischemic injury (HIE). This cohort of neonates, all of whom underwent therapeutic hypothermia, were evaluated with Near Infrared Spectroscopy (NIRS) to obtain the hemoglobin volume index (HVx) as a marker of cerebral autoregulation that identifies the optimal mean arterial blood pressure (MAPOPT) at which optimal autoregulation is achieved. The investigators identified the MAPOPT for 3 time periods: hypothermia, rewarming and first 6 hours of normothermia. They compared the effect of MAPOPT on neurodevelopmental outcomes at 2 years. A total of 19 patients remained in the study after follow up. Within the cohort of 19 patients, only 15 patients underwent the full research battery of standardized cognitive and motor scales. Neurodevelopmental outcomes were classified as impaired or unimpaired. In this cohort, 42% of the children were identified as having impaired cognitive or motor function. The investigators only found differences in outcome when they compared the MAPOPT during the rewarming period. The investigators found that children with an impaired outcome had higher MAPOPT (p=0.023), and spent a greater percentage of time during rewarming with blood pressures under their identified MAPOTP (p=0.048). The authors suggest that monitoring and management of HVx as a marker of autoregulation may have an impact on neurodevelopmental outcomes of neonates affected by HIE. [1]

COMMENTARY. HIE in the neonate remains a public health concern. Therapeutic hypothermia is currently the standard of care for neonates with moderate to severe HIE, and although it has decreased death and disability, adverse neurodevelopmental outcomes are still prevalent [2]. The mechanisms of cerebral perfusion patterns in neonates are not well understood [3]. Understanding patterns of cerebral blood flow and autoregulation is paramount to establish therapies directed at maintaining adequate cerebral perfusion and avoid reperfusion injury. Although NIRS monitoring does not provide a direct prognostic utility in the management of infants with HIE undergoing hypothermia [4], it may provide a putative value for autoregulation and optimal blood pressures goals, and guide management to achieve adequate cerebral perfusion in the neonate.

This study highlights the need for closer hemodynamic monitoring aimed at maintaining optimal cerebral autoregulation in neonates undergoing therapeutic hypothermia as this may impact neurodevelopmental outcomes.
